# Pathophysiology of Chylous Anasarca Caused by Lymphatic Occlusion: A Case Report and Review of the Literature

**DOI:** 10.3390/jpm15060216

**Published:** 2025-05-26

**Authors:** Antoine Mathivet, Martin Bertrand, Isabelle Quere, Jean-Christophe Gris, Julien Ghelfi, Julien Frandon

**Affiliations:** 1 Centre Hospitalier Universitaire Nîmes, Place du Pr R. Debré, 30029 Nîmes, France; 2 Department of Medicine, Université de Montpellier, 163 Rue Auguste Broussonnet, 34090 Montpellier, France; 3 Centre Hospitalier Universitaire de Montpellier, 191 Av. Doyen Gaston Giraud, 34090 Montpellier, France; 4 Department of Radiology, Grenoble-Alpes University Hospital, 38700 Grenoble, France

**Keywords:** thoracic duct, lymphatic drainage, lymphatic disorders, lymphatic system, lymphatic occlusion, chylous leakage

## Abstract

**Objective:** The aim of this study was to propose a pathophysiological hypothesis for the occurrence of non-traumatic chylous effusions and Central Conducting Lymphatic Anomalies (CCLAs) related to lymphatic occlusion. **Methods**: We investigated the case of a 39-year-old woman managed at Nîmes University Hospital for chylous anasarca related to an endoluminal lymphatic occlusion. We then conducted a comprehensive review of the literature on CCLAs. **Results**: Lymphatic drainage is a dynamic process. Obstacles to lymphatic drainage via the thoracic duct can lead to chylous anasarca, depending on where the obstruction is. Lymphatic occlusion seems to be an explanation for certain CCLAs. **Conclusions**: Understanding CCLAs via the theory of lymph occlusion opens the way to new therapeutic options, but requires further investigation in order to personalize the patient’s treatment.

## 1. Introduction

Central Conducting Lymphatic Anomalies (CCLAs) or Central Conducting Lymphatic Disorders (CCLDs) are rare conditions affecting the thoracic duct and its major tributaries [1,2]. They are poorly described in the literature [1]. These anomalies are characterized by dilated, tortuous lymphatic channels, causing lymphatic stasis and reflux. They are responsible for anomalies in the flow of chyle, causing its leakage into the peritoneal, pleural or pericardial spaces, diffuse lymphoedema, chylorrhoea, protein-losing enteropathy and various other symptoms [1,2,3,4,5].

Although the pathophysiology of CCLAs remains unclear, many etiologies have been suspected and reported: when it is neither traumatic, infectious nor tumor-related, chylous anasarca can be related to lymphatic drainage anomalies via the thoracic duct [6,7,8]. These anomalies are suspected to be obstructive or functional [2]. Obstructive causes are not always well-characterized and may be due to extraluminal compression, malformation or iatrogenesis, but often remain idiopathic [3,6]. As pathophysiology and etiologies remain unclear, CCLAs are often under-diagnosed and thus under-treated, exposing patients to long-term complications. The consequences can be severe: electrolyte disturbances, malnutrition, infections and even death (30–50% mortality after untreated chronic chylothorax) [9].

Our objective is to propose a pathophysiological hypothesis for the occurrence of non-traumatic chylous effusions by discussing a clinical case encountered at Nîmes University Hospital, combined with a comprehensive review of the literature. This could open the way to new therapeutic solutions and avoid the complications of the pathology.

## 2. Case Report

**Figure 1:**
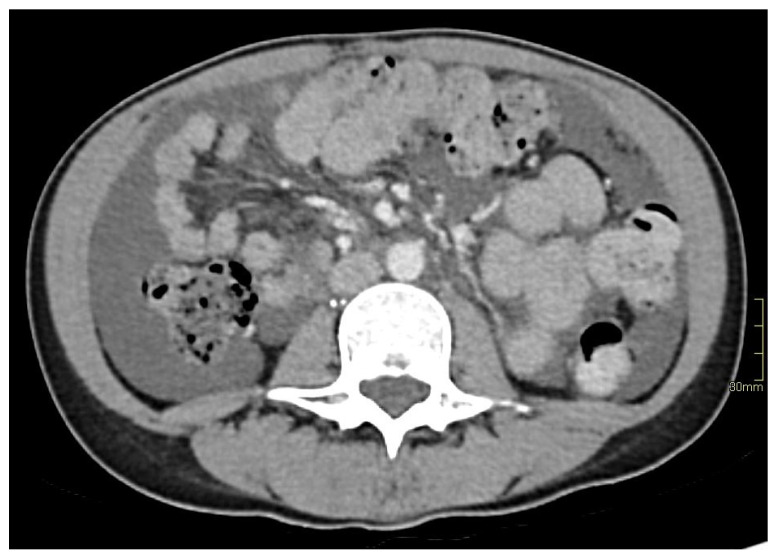
Initial tomodensitometry showing abundant ascites.

We hereby report the case of a 39-year-old North African woman with no relevant personal or familial medical history who presented with chylous ascites, which had appeared in the first trimester of her first pregnancy in 2013 at the age of 29 (Figure 1). Before pregnancy, she was totally asymptomatic. Ascites persisted after her uncomplicated vaginal delivery and progressively worsened, requiring a low-fat diet and increasingly frequent drainage every 1 to 2 months, evacuating 6 L of ascites every time. These treatments, combined with octreotide administration, did not improve ascitic effusion. Biochemical analysis of the ascites revealed a high level of triglycerides (about 30 mmol/L) with chylomicrons. She had never had ascites before. Several examinations (thoraco-abdominal-pelvic CT scan, myelogram, abdominal-pelvic MRI and PET scan) were made, none of which revealed any specific pathology that might explain the clinical picture. An exploratory abdominal laparoscopy with biopsies ruled out peritoneal tuberculosis but identified a lesion due to peritoneal endometriosis. An abdominal lympho-MRI showed a generally dilated retroperitoneal lymphatic system with no anomalies in the thoracic duct. In this context, the patient was referred to Nîmes University Hospital for further exploration in June 2023.

**Figure 2:**
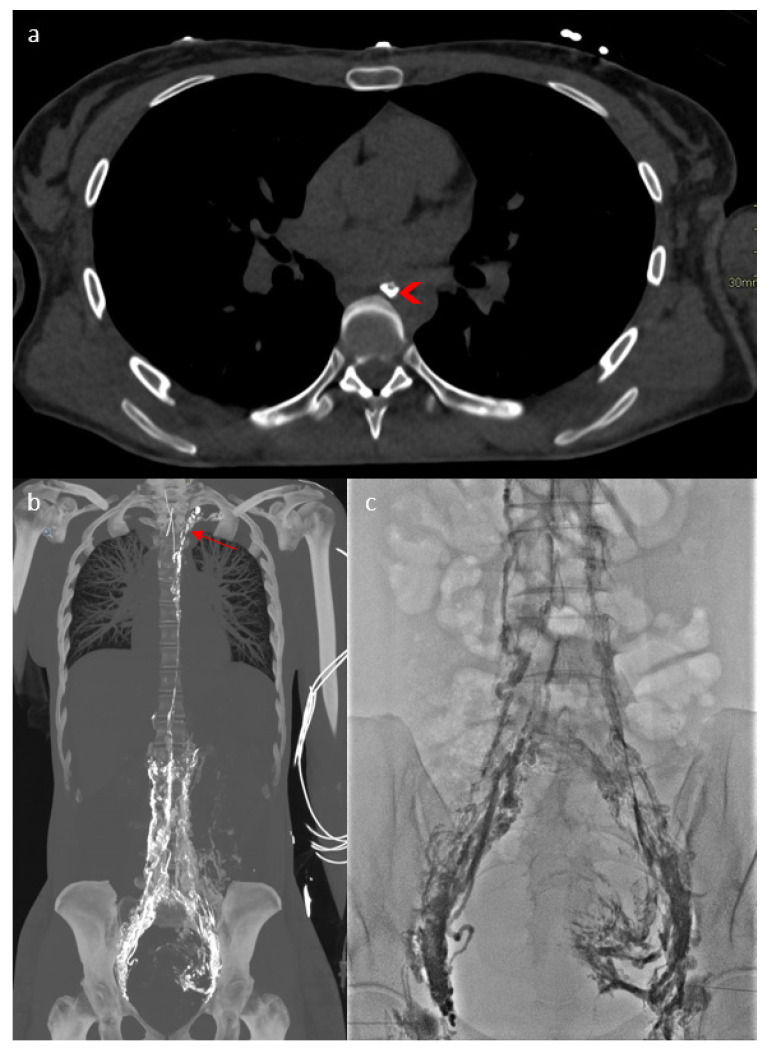
Four-dimensional CT lymphography showing the thoracic duct ((**a**)—arrowhead), diffuse involvement of the lymphatic network (**b**) with a “Medusa head” (arrow) aspect of the thoracic duct and lipiodol reflux to the pelvis (**c**).

In addition to chylous ascites, the initial explorations by thoracic CT and MRI revealed a thoracic duct terminating at the left jugulo-subclavian angle. As an obstruction was suspected, lymphography was performed in the 4D-CT room in September 2023, combining a CT scan and a flat-panel detector for multimodal management with real-time fluoroscopic imaging to assess the dynamic function of the lymphatic pathways, and intraoperative CT for precise 3D anatomy (Figure 2a). This involves mapping the lymphatic system by injecting lipiodol via inguinal node puncture and lymphography, and retrograde catheterization with dynamic contrast injection in the thoracic duct to allow dynamic assessments. This revealed diffuse involvement of the lymphatic network (Figure 2b) with lipiodol reflux in the pelvis (Figure 2c). The thoracic duct appeared to be plexiform, atrophic and hypoplastic, with a “medusa head” appearance (Figure 2b). Dynamic analysis showed multiple stenoses of the thoracic duct obstructing lymphatic return, and a lymphatic reflux in the abdomen by hyperpression of the small lymphatic vessels because there was no visible injury, no localized leakage and no external compression.

**Figure 3:**
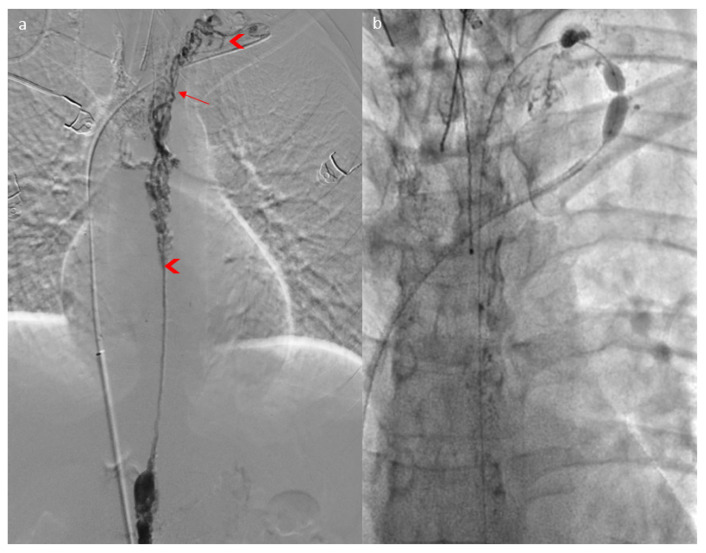
Lymphography (**a**) showing a “Medusa head” aspect of the thoracic duct (arrow) and two stenoses of the thoracic duct (arrowhead), which were impossible to dilate through angioplasty (**b**).

It was decided to perform thoracic duct angioplasties (Figure 3a): gentle angioplasty, using a 5mm balloon at 2 bars of the proximal third of the thoracic duct judged stenosed, and higher-pressure angioplasty of the short diaphragmatic stenoses in the cervical portion with the same balloon, which was moved. However, two stenoses persisted and were impossible to dilate despite pressures over 20 bars and several angioplasty balloon ruptures (Figure 3b) at the exit of the mediastinum and at the lympho-venous junction (Figure 3a).

**Figure 4:**
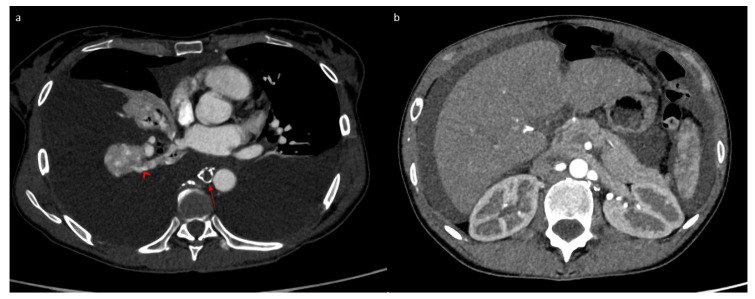
Tomodensitometry showing an abundant bilateral chylothorax (**a**) and a small amount of ascites (**b**). We noticed a dilated thoracic duct with a lacunar aspect evoking an obstruction process (arrow) and lipiodol reflux into the lungs (arrowhead).

**Figure 5:**
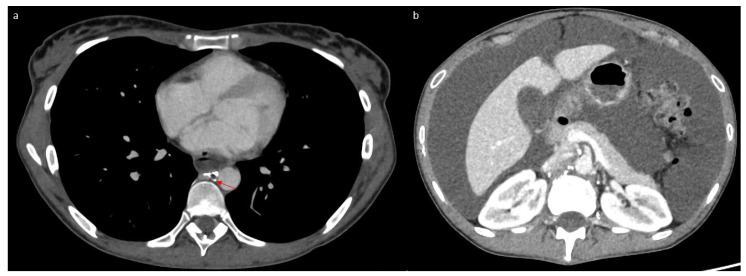
Tomodensitometry showing complete resolution of the pleural effusion (**a**) with abundant ascites (**b**). We noted a non-dilated thoracic duct with a lacunar aspect (arrow).

Following this procedure, the patient returned home with outpatient care. She presented a week later to the emergency department with progressive, then severe, respiratory distress and orthopnea. Thoracic CT revealed abundant bilateral pleural effusion (Figure 4a). Only a small amount of ascites was present (Figure 4b). A dilated lacunar structure appeared in the middle of the “Medusa head” image, indicating native thoracic duct congestion downstream from a possible truncal occlusion (Figure 4a). The bilateral chylothorax was accompanied by lipiodol reflux from the lymphography into the peribronchial area, also confirming lymph reflux into the lung (Figure 4a). A left thoracic drain was placed, allowing the evacuation of 6 L of chylothorax. Biochemical analysis of the pleural liquid revealed a high level of triglycerides (27.62 mmol/L) with chylomicrons. The patient quickly improved, with the drain soon drying up and being removed after 3 days. The chylothorax resolved completely within 10 days, as confirmed by follow-up CT (Figure 5a). However, simultaneously, the chylous ascites significantly worsened, becoming very abundant (Figure 5b) and requiring regular drainages again, with high triglyceride levels (13.35 mmol/L) and chylomicrons. This time, the native thoracic duct appeared much less dilated (Figure 5a).

The patient was initially treated as if she had a malformative anomaly, but CCLAs were diagnosed, so the treatment was reassessed. After failure of medical management (dietetic and sirolimus) and subsequent interventional treatment through thoracic duct angioplasties, several options remained feasible for this patient. Indeed, she now depends on weekly repeated ascitic paracentesis, and her quality of life is significantly impaired. Possibilities include a Denver peritoneo-jugular shunt to redirect the lymphatic flow into the venous system, as well as a lympho-venous anastomosis procedure, either surgically or through interventional radiology, although this procedure has rarely been performed and is still in the experimental phase. In any case, the most appropriate therapeutic option for this rare condition should be discussed at a multidisciplinary team meeting with specialists of the lymphatic system.

## 3. Discussion

### 3.1. Case Discussion and Physiopathological Hypothesis

**Figure 6:**
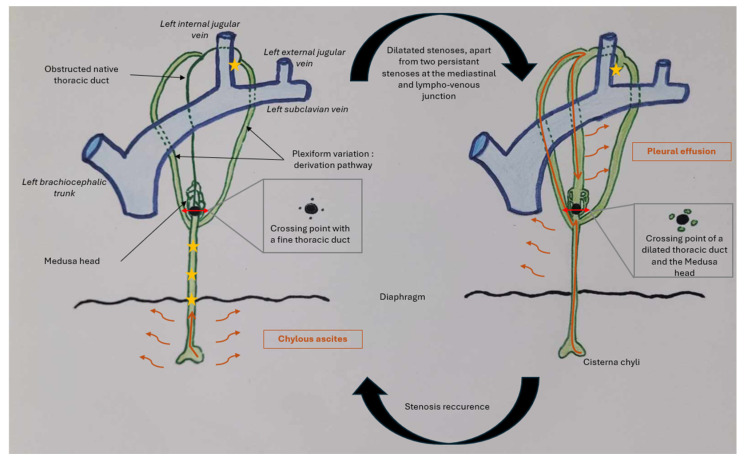
Diagram that shows lymphatic flow in the patient throughout her clinical history according to our hypothesis. Lymphatic flow traveling through the thoracic duct is represented by the orange arrow, and lymphatic effusion by the small orange wavy arrows. The stars represent stenoses. The large black dot represents the point where the native thoracic duct is obstructed, and the double red arrows represent sections of the thoracic duct as shown above.

We hereby report the case of a 39-year-old woman who has been experiencing chylous ascites for over 10 years, with multiple stenoses of the thoracic duct. She presented symptoms progressively during pregnancy. Before pregnancy she had no symptoms, raising the question of the triggering factor. During pregnancy, modification of Virchow’s triad leads to an increase in thromboembolic events, which could possibly also affect the lymphatic system and explain an acquired lymph occlusion. Dilating these thoracic duct stenoses, apart from one stenosis at the exit of the mediastinum and one at the lympho-venous junction, resulted in a reduction in chylous ascites but led to the development of chylothorax. At this stage, the native thoracic duct was dilated, appearing as a lacunar image in the center of the dilated lymphatic collateral pathways marked with lipiodol, indicating segmental obstruction of the thoracic duct. The patient then experienced a recurrence of the stenosis of the proximal third of the thoracic duct because angioplasty without stenting is sometimes ineffective. When this proximal stenosis recurred, massive chylous ascites reappeared at the same time that the chylothorax disappeared, and the initial symptomatology recurred. This was because most of the lymph originated from abdominal drainage, and the thoracic duct was only handling lymphatic return from the lungs and the left upper limb at a very low flow rate, with more fluid lymph without chylomicrons. This sequence is illustrated in Figure 6.

Lymphatic drainage is a dynamic process. When stenosis is present in the lower thoracic duct, chyle—mainly produced by the lower part of the body—accumulates in the abdomen. The smaller amount of chyle produced by the thorax can still flow and does not cause chylous effusion. Furthermore, the lymph has a different composition, with thick, milky lymph from the abdomen (high levels of triglycerides and chylomicrons) and more fluid lymph elsewhere. However, when the stenoses are relieved, chyle can progress through the thoracic duct, but the persistence of a non-dilatable stenosis at the lympho-venous junction leads to its accumulation in the pleura, after it has taken alternative pathways through other branches of a plexiform thoracic duct.

The presence of a non-dilatable stenosis in the thoracic duct may correspond to the result of an occlusive phenomenon. In certain situations, plexiform variations of the thoracic duct (found in the literature in about 10% of cases [10]) might correspond to varicose bypass pathways in response to obstruction of the native thoracic duct. Given the image visualized, this obstruction could be related to a process similar to that of a thrombotic phenomenon, but the thrombotic properties of lymph remain to be explored [11]. This might be supported by the hematological changes leading to an increase in thromboembolic events during pregnancy. It could also support the hypothesis that bypass pathways are formed following thoracic duct obstruction, preventing drainage into the venous system, just as varicose veins develop in cases of venous thrombosis.

Although cases of thoracic duct obstruction due to malformations, tumors or external compression have been described in the literature [12], this particular case highlights a true lymphatic occlusion. On the imaging, the patient presented an endoluminal obstruction resembling a thrombus, and a “Medusa head” appearance of the thoracic duct, suggestive of varicose lymphatic vessels. This specific presentation was distinct from previously described cases of lymphatic obstruction, where the cause was often extrinsic compression or structural malformation. Our findings emphasize the importance of considering lymphatic occlusion in the event of persistent chylous effusion, particularly when no other cause can be identified. Furthermore, this case provides new insights into the pathophysiology of the condition and emphasizes the potential for innovative therapeutic approaches, such as evaluating anticoagulant therapies for “lymphatic thromboses” and exploring lympho-venous anastomoses, as promising treatment strategies for patients with this rare condition.

### 3.2. Comprehensive Review of the Literature About CCLA Etiologies and Therapeutic Strategies

#### 3.2.1. Definition

Central Conducting Lymphatic Anomalies (CCLAs) and Central Conducting Lymphatic Disorders (CCLDs) are rare, little-known conditions, with a large variety of symptoms [1,2,3,4,5,13]. They lack diagnostic criteria and are thus under-diagnosed, leading to major complications [5]. The diagnosis is established based on a convergence of findings, including the clinical history and examination, biological analysis of any effusions and imaging results [5,13].

#### 3.2.2. Etiologies

Many explanations for CCLAs have been suggested, and some authors [1,2,3,4,5] report that CCLAs are due to the following:

- Congenital obstructions, with a dysplastic thoracic duct or stenosis of the lympho-venous junction; many advances have been made in this domain, and genetic mutations have been identified [5].
- Acquired obstructions, with accidental thoracic duct ligation during thoracic surgery.
- Functional anomalies, with high central venous pressure preventing lymphatic drainage from the thoracic duct into the venous system.

In a retrospective study on 11 patients, Srinivasan et al. [12] identified several causes of thoracic duct obstruction. Stenosis of the thoracic duct outlet was the most common, followed by extrinsic compression, thoracic duct outlet occlusion and thoracic duct ligation. The nature of the thoracic duct outlet occlusion was not detailed precisely. For Chen and Itkin [6], the etiology of non-traumatic chyle leakage was mostly idiopathic, highlighting the lack of data on CCLAs.

In 2023, the lymph occlusion theory was raised in a publication by Ghelfi et al. [14]. Several cases of patients were reported, one of whom presented with decompensated edematous-ascitic cirrhosis, possibly due to a pseudo-thrombotic obstructive phenomenon of the thoracic duct. They formulated the hypothesis that a thrombotic phenomenon in the thoracic duct might be responsible for an increase in lymphatic pressure and, hence, upstream portal pressure, and therefore decompensated edematous-ascitic cirrhosis. This seems to corroborate the hypothesis that lymph occlusion might be a plausible physiological explanation for chylous anasarca presentations when they are neither iatrogenic nor malformative.

#### 3.2.3. Imaging

Diagnoses are largely based on imaging findings, and a great deal of progress has been made in the field of lymphatic imaging. The thoracic duct is a structure with considerable anatomical variability, and typical anatomy is only observed in 40–65% of individuals [15]. Variations are found in every portion of the thoracic duct [16]: cisterna chyli [17,18], the diaphragmatic region [19], the thoracic portion [9,10,15,20,21], the cervical portion and the lympho-venous junction [22,23,24,25,26]. High-performance imaging modalities are required to evaluate individual thoracic duct anatomy and each patient’s pathology to personalize their treatment and intervention.

Magnetic Resonance Imaging (MRI) performs well in the field of lymphatics. T2-weighted sequences [27,28], with fat signal suppression, offer satisfactory detection of the thoracic duct. Specialized lymphatic sequences, constituting lympho-MR, seem to be the best means of imaging for studying the anatomy and pathologies of the thoracic duct [29,30]. Interventional imaging can also be performed with 4D-CT lymphography, as depicted in this case report, showing the real-time dynamic process of lymphatic drainage. Both imaging modalities can lead to the adaptation of management and interventions for each specific situation.

#### 3.2.4. Treatments

In the context of CCLAs and CCLDs responsible for chylous anasarca, new therapeutic approaches are emerging, though their efficacy remains variable and their indications are still not clearly defined. It is essential to categorize thoracic duct drainage disorders to better understand their pathophysiological mechanisms and identify the most effective treatments for patients.

The first stage of treatment is nutritional management. Patients with chronic CCLAs often suffer from malnutrition due to protein and fatty acid loss. Eliminating all lipid intake can reduce chyle flow by a factor of ten, requiring a strict diet or exclusive enteral or even parenteral nutrition. This diet includes the use of medium-chain triglycerides, as fatty acids with fewer than ten carbon atoms are absorbed directly into the portal venous system without passing through the lymphatic system [7,9,31]. Patients are also treated with octreotide, a somatostatin analogue that reduces chyle flow [7,9,31]. Octreotide is often effective but should be used with caution, particularly in neonates and infants, as it can cause necrotizing enterocolitis [32]. Some patients may also be treated with an mTOR inhibitor, sirolimus, which inhibits a serine–threonine kinase in the PI3K/AKT signaling pathway. This pathway is overactivated in most lymphatic malformations and certain central lymphatic anomalies [13]. However, this approach, combined with repeated drainage procedures, may improve symptoms but does not resolve the underlying problem. Given the previously mentioned consequences of chronic chylous anasarca and the associated impairment in quality of life, it is crucial to develop effective therapeutic options.

New interventional radiology procedures to deal with these conditions are gradually emerging. These involve interventions on the thoracic duct via anterograde cannulation (through abdominal lymphatic vessels and inguinal nodes) or retrograde access (via the venous system through the lympho-venous junction) [15] and require a precise assessment of the patient’s lymphatic anatomy. In cases of traumatic chylous effusions, for several years now, thoracic duct embolization has been performed as an alternative to surgical ligation, particularly post surgery, with moderate efficacy [6,33,34]. However, this may be beneficial in select cases. Recently, Ghelfi et al. [35] introduced thoracic duct stenting as a new approach to managing refractory ascites in cirrhotic patients who are not eligible for transjugular intrahepatic portosystemic shunt insertion, representing a potentially promising therapy for CCLAs.

Surgical management may also be considered for the treatment of CCLAs, particularly in cases for which conservative treatment fails. Thoracic duct ligation, an alternative to embolization, can be used to prevent chyle leakage into the pleural or peritoneal spaces. Lympho-venous anastomosis has been proposed as a promising approach aimed at restoring functional lymphatic drainage into the bloodstream, but has mostly been performed at the cervical level for thoracic duct outlet stenosis or obstruction [12,36,37,38,39,40,41,42,43,44,45,46,47,48]. In the case of a mediastinal obstruction, as in the case presented here, lympho-venous anastomosis should be performed at a different site. Peritoneovenous shunting can also be used in certain cases to redirect chyle into the systemic circulation via an intra-abdominal pathway [2,4]. However, given the low prevalence of the disease, no guidelines currently define the role of the various treatment options, and further investigations are needed.

These various treatments, some of which remain poorly evaluated, require precise lymphatic mapping to determine the underlying etiology and, when applicable, to locate the exact site of the obstruction or leakage. The appropriate therapeutic strategy must be discussed in a multidisciplinary team meeting with specialists of the lymphatic system in order to personalize treatment.

## 4. Conclusions

In discussing the case of this patient, who was referred to Nîmes University Hospital, we proposed a pathophysiological hypothesis to explain certain CCLAs. A lymphatic occlusion caused by a tortuous, dilated “Medusa-head”-like aspect of the thoracic duct could be responsible for chylous anasarca. Understanding Central Conducting Lymphatic Anomalies (CCLAs) through the lens of the lymphatic occlusion theory opens new avenues for therapeutic interventions. This perspective encourages further research into personalized treatment strategies, such as the evaluation of anticoagulant therapies and the exploration of lympho-venous anastomoses. These emerging therapeutic options are essential for improving patient outcomes in cases of rare lymphatic disorders. We believe this study contributes to a deeper understanding of the pathophysiology of chylous anasarca and offers promising prospects for future research.

## Data Availability

The original contributions presented in this study are included in the article. Further inquiries can be directed to the corresponding author.

## Abbreviations

The following abbreviations are used in this manuscript:

4D-CT	Four-Dimensional Computed Tomography
CCLAs	Central Conducting Lymphatic Anomalies
CCLDs	Central Conducting Lymphatic Disorders
